# Piloting Laboratory Quality System Management in Six Health Facilities in Nigeria

**DOI:** 10.1371/journal.pone.0116185

**Published:** 2014-12-26

**Authors:** Henry Mbah, Emmanuel Ojo, James Ameh, Humphrey Musuluma, Olubunmi Ruth Negedu-Momoh, Feyisayo Jegede, Olufunmilayo Ojo, Nkem Uwakwe, Kingsley Ochei, Michael Dada, Donald Udah, Robert Chiegil, Kwasi Torpey

**Affiliations:** Family Health International (FHI360), Plot 1073-A1, GODAB Plaza, Area 3, Garki-Abuja, Nigeria; Tulane University School of Public Health and Tropical Medicine, United States of America

## Abstract

**Background:**

Achieving accreditation in laboratories is a challenge in Nigeria like in most African countries. Nigeria adopted the World Health Organization Regional Office for Africa Stepwise Laboratory (Quality) Improvement Process Towards Accreditation (WHO/AFRO– SLIPTA) in 2010. We report on FHI360 effort and progress in piloting WHO-AFRO recognition and accreditation preparedness in six health facility laboratories in five different states of Nigeria.

**Method:**

Laboratory assessments were conducted at baseline, follow up and exit using the WHO/AFRO– SLIPTA checklist. From the total percentage score obtained, the quality status of laboratories were classified using a zero to five star rating, based on the WHO/AFRO quality improvement stepwise approach. Major interventions include advocacy, capacity building, mentorship and quality improvement projects.

**Results:**

At baseline audit, two of the laboratories attained 1- star while the remaining four were at 0- star. At follow up audit one lab was at 1- star, two at 3-star and three at 4-star. At exit audit, four labs were at 4- star, one at 3-star and one at 2-star rating. One laboratory dropped a ‘star’ at exit audit, while others consistently improved. The two weakest elements at baseline; internal audit (4%) and occurrence/incidence management (15%) improved significantly, with an exit score of 76% and 81% respectively. The elements facility and safety was the major strength across board throughout the audit exercise.

**Conclusion:**

This effort resulted in measurable and positive impact on the laboratories. We recommend further improvement towards a formal international accreditation status and scale up of WHO/AFRO– SLIPTA implementation in Nigeria.

## Background

In Nigeria, clinical laboratories are grossly inadequate with poor infrastructure [1] and Quality Management Systems (QMS) are uncommon [2]. Family Health International 360 (FHI360), with funding from the President's Emergency Plan for AIDS Relief (PEPFAR) through United States Agency for International Development (USAID) and Global Fund to fight AIDS Tuberculosis and Malaria (GFATM) supported the Government of Nigeria to scale up Antiretroviral therapy (ART) services to 134 health care facilities across 36 states and the Federal Capital Territory (FCT) from 2005 to 2012. Scaling-up ART service includes laboratory strengthening and support aimed at providing quality services to HIV/AIDS patients.

Access to services with the highest standards remain the major focus in FHI360 ART program implementation. Although this intervention led to improve laboratory service, over time it became apparent that this level of capacity did not translate to quality laboratory services as defined by some international standards for clinical laboratories, including ISO 15189 [3]. Thus, beside investment in the expansion of diagnostic access, concurrent improvements and more attention in the quality of laboratory testing are needed to ensure clinician and patient confidence in test results [4]. Accreditation is emerging as a preferred framework for building quality medical laboratory systems in resource-limited settings [4]. Accreditation symbolizes verification that laboratories are adhering to established quality and competence standards deemed necessary for accurate and reliable patient testing and the safety of staff and the environment [5]. Achieving accreditation in laboratories is a challenge in Nigeria like in most developing countries. Overall, the quality of medical laboratory service in Africa as judged by accreditation is much lower than would be desired. A May 2013 survey indicated that over 91% of the 340 accredited laboratories in sub-Saharan Africa were in South Africa and 37 of 49 countries had no laboratories accredited to international quality standards [6] The need to increase support to strengthen and improve on the quality of medical laboratory services in the rest of Africa is paramount.

To trigger and coordinate efforts to improve on laboratory service quality, the World Health Organization, Regional Office for Africa (WHO-AFRO) in collaboration with other stakeholders developed and launched a novel laboratory accreditation approach in Kigali, Rwanda in 2009 [7]. This effort led to the Strengthening Laboratory Management Towards Accreditation (SLMTA) training program [8] and Stepwise Laboratory (Quality) Improvement Process Towards Accreditation (SLIPTA) tools [9]. This program intends to provide an interim pathway for measuring, monitoring and recognizing improvement toward the realization of international laboratory standards and subsequent application to a national or internationally recognized accreditation body [9]. Member states were encouraged to implement this approach to ensure quality improvement in laboratory services. In Nigeria, SLMTA program implementation was piloted in 23 PEPFAR supported health facilities. With the successful implementation of this process; it is believed that there will be measurable quality improvement in medical laboratory services delivery and consequently on the entire healthcare system across the nation. This improvement will later be measured through the WHO/AFRO– SLIPTA audit and based on performance some of the sites will be supported for international accreditation. Six of these 23 facilities in the pilot program in Nigeria are supported by FHI360. Here we report on progress of these six public health facility laboratories.

## Methodology

### Project design and setting

This was a quantitative longitudinal audit. Implementation of the in-country SLMTA program [8] was piloted in six health facility laboratories supported by FHI360 across five states in Nigeria. Five out of the six laboratories were stand-alone ART laboratories. The sixth, General Hospital (GH) Lagos, operates as a fully integrated health system [10] where ART related laboratory services are embedded into the general laboratory outfit. (Table 1).

**Table 1:** Site Description.

Site name	FMC Owerri	GH Lagos	GH Calabar	DLHMH Calabar	CH Benin	IDH Kano
Location (Town)	Owerri	Lagos Island, Lagos	Calabar	Calabar	Benin City	Kano
State	Imo	Lagos	Cross River	Cross River	Edo	Kano
Coordinates	5.4850° N, 7.0350° E	6.4531° N, 3.3958° E	4.9500° N, 8.3250° E	4.9500° N, 8.3250° E	6.3176° N, 5.6145° E	12.0000° N, 8.5167° E
Level of operation	Tertiary	Secondary	Secondary	Secondary	Secondary	Secondary
Affiliation	Government	Government	Government	Government	Government	Government
ART commencement date	February 2008	February 2007	April 2005	September 2007	September 2005	April 2005
Laboratory setup	Standalone ART laboratory	Fully integrated ART laboratory service within 6 departments	Standalone ART laboratory	ART and MDR –TB BSL-3 laboratory	Standalone ART laboratory	Standalone ART laboratory
# of laboratory Personnel*	7 (LS = 3, LT = 2, CL = 2)	39 (LS = 17, LT = 13, LA = 4, CL = 5)	22 (LS = 10, LT = 2, LA = 8, CL = 2)	11 (LS = 6, LT = 1, LA = 6, CL = 1, FE = 1)	4 (LS = 3, CL = 1)	16 (LS = 9, LT = 1, LA = 4, CL = 2)
Average # of samples tested per month	225	1,435	300	284	450	258
Current # of ART enrolled patients as at 2013 March	5,489	3,970	1,948	653	4,263	4,468

FMC: Federal Medical Center Owerri; GH: General Hospital Lagos; GH: General Hospital Calabar; DLHMH: Dr Lawrence Henshaw Memorial Hospital, Calabar; CH: Central Hospital Benin; IDH: Infectious Disease Hospital Kano, #: number, ART- Antiretroviral Treatment, MDR-TB, Multidrug Resistant Tuberculosis, BSL: Bio-Safety level; LS: Laboratory Scientist, LT:–Laboratory Technician, LA: Laboratory Assistant, CL: Cleaner, FE: – Facility Engineer.

*Reflects personnel status at baseline audit only.

### Advocacy and Sensitization of Stakeholders

To ensure ownership and sustainability of the laboratory accreditation program, a one day sensitization meeting was organized in March 2010 by FHI360 in collaboration with the Medical Laboratory Science Council of Nigeria (MLSCN). Key stakeholders include; State Commissioners for Health, facilities Chief Medical Directors, head of laboratories, FHI360 zonal managers, representatives from Federal Ministries of Health and representatives of donor agencies. The concept, benefit, role of the various parties and the road map for accreditation were presented and discussed. A follow up meeting in November 2010 was used to formally present the reports of the baseline audit, discuss the gaps, redefine strategies, assign responsibilities and advocate for resources and commitment towards the accreditation process. At the facility level, outcome of audit exercise and deficiencies were disseminated immediately by the team of auditors to the hospital management and administrators. This meeting also serves as an advocacy forum on the required support to further the accreditation process.

### Trainings and Workshops

- The first step to support the national effort by PEPFAR Nigeria was the training of six in-country officers known as SLMTAns/SLMTA Auditors on SLMTA [8] concepts, procedure and field application organized by Center for Disease Control (CDC) Atlanta, USA Global AIDS Program (GAP) in February 2010 in South Africa for 2 weeks.
- Practical implementation of Quality System Management (QSM) training was conducted in collaboration with Royal Tropical Institute (KIT) Biomedical Consultants, Netherlands in March 2010. A total of 24 participants including facility staff and FHI360 personnel were trained for 5 days.
- Phase 1, 2 and 3 of SLMTA workshop [8] was held in Lagos; November 2010, Zaria; September 2011 and Abuja; July 2012. A total of 21 facility staff from FHI360 supported sites participated. The SLMTA workshop is unique in its approach and featured; task based curriculum with lots of activity, the training content was closely linked with the WHO AFRO checklist [11] with emphasis on improvement projects. The modules covered in the SLMTA curriculum are: productivity management, work area management, inventory management, procurement management, equipment management and maintenance, Quality assurance, specimen management, laboratory testing, test result reporting and documents & records.
- QSM and ISO 15189 training was conducted in collaboration with FHI360 regional laboratory team in January/February 2011 for 50 persons including facility and FHI360 technical staff. Some areas for accreditation not adequately addressed by SLMTA were covered like quality control principles and practices, writing standard operating procedures, biologic safety, and quality assurance manager training.
- FHI360 laboratory accreditation committee was formed comprising mainly facility quality officers and FHI360 state technical officers. The team met in a 5 day workshop to develop, review and harmonize policies, Standard Operating Procedures (SOPs), templates, worksheets, forms, quality and safety manual in compliance to the requirements of ISO 15189 standards.
- All the trainings were further stepped down at the facility level, to other members of the laboratory team by facility staff that benefited from central trainings, coordinated by the accreditation committee. The trainings contributed significantly to the marked quality improvement recorded in all the facilities as each training was able to address different areas of concern and challenge facing SLMTA implementation.

### Laboratory Assessments, Mentorship and Improvement Projects

Baseline and follow up assessments were conducted by the in-country SLMTA auditors between April to August 2010 and July to August 2011 respectively. The exit audit was conducted by a mixed team of in country SLMTA auditors and CLSI auditors from December 2012 to February 2013. The WHO/AFRO– SLIPTA audit checklist [11] was used. The sum of the score on the checklist was expressed as percentage of a total obtainable score of 250. The facility quality status was determined by using a zero to five star rating, calculated as follows: less than 55% = 0-star, 55 to 64% =  one-star, 65 to 74%;  = 2-star, 75 to 84%;  = 3-star, 85 to 94% = 4-star and 95% and above  = 5-star [9]. Best known to the hospital management, two departments (Parasitology and Blood Transfusion Services) of the intergraded laboratory, General Hospital, Lagos were not presented for baseline assessment but participated in both the follow up and exit audits. Each visit provided the auditors the opportunity to mentor the sites and advise them on how to improve on their current standard of QMS. After an audit exercise, gaps and technical details were discussed with the laboratory team. The trainings and workshop was supported with targeted mentorship program by FHI360 Lab Quality Assurance (QA) team including a SLMTA certified mentor. On-site mentors provided the opportunity for laboratory personnel to translate knowledge acquired during the training to real laboratory implementation. The objectives of the mentorship exercise were to; evaluate improvement projects, work with the laboratory team to understand the usage of the newly developed QMS documents and train personnel on answering audit questions. This exercise included group and one-on-one training on various policies in the quality manual, key SOPs, laboratory safety and utilization of various forms to capture quality and technical records.

Internal post-mentorship assessment was conducted by utilizing the WHO/AFRO– SLIPTA audit checklist in order to measure improvement after addressing baseline gaps and the impact of the mentorship program. Series of improvement projects were provided to participants following each training especially the SLMTA workshop. The projects were chosen based on the various gaps identified during the previous audits. Some of the improvement projects executed at all sites are highlighted in Table 2. Progress and goal completion were measured using the quality improvement model, though some of the projects were only documented and displayed as a one off activity. For example, redesigning the floor plan of the laboratory to improve efficiency and hence increase productivity. Through mentorship, the laboratory teams incorporated a schedule involving every staff member that include weekly meetings, quizzes and exercises, quality indicator monitoring, individual contributions to standard operating procedure development, etc.

**Table 2:** Major improvement projects to address identified gaps.

Improvement project	Objective(s)	Means of verification
Redesigning the Laboratory floor plan.	To achieve optimal productivity, improve on workflow and safety	Pre and post improvement pictures.
Implementation of staff meeting	To achieve optimal productivity through effective communication/discussion	Meeting notes with appropriate content.
Implementation of fire drill exercise	To increase fire response awareness and consequently safety.	Fire drill records, observe response time during drills.
Implementation of customer satisfaction survey	To fulfil customer desire and improve customer focus process.	Record of survey analysis with evidence of intervention.
Creation of personnel files	To ensure availability of complete personnel records for administrative and audit purposes	Complete personnel files with required documents
Implementation of internal audit system.	To fulfil ISO standard and assist in identifying non-conformities and OFIs.	Well utilized audit checklist and audit records
Implementation of duty roster.	To improve personnel efficiency and consequently productivity.	Copy of approved and utilized lab duty roster.
Selection and monitoring of quality indicators e.g. TAT of releasing results	To track a particular area of laboratory process	Availability and analysis of quality indicator monitoring records

OFI- opportunity for improvement; ISO- International Organization for Standardization; TAT- Turnaround time.

### Data Analysis

We conducted descriptive analysis of the data. The average performance based on the Quality System Essentials (QSE) were computed for the six laboratories and compared at baseline, follow up and exit audit. In addition, the relative change in QSE scores at baseline, follow up and exit audit was calculated for each laboratory. Within each section of the WHO–AFRO SLIPTA checklist, the average performance of the five laboratories was computed. From the total percentage score obtained, the quality status of laboratories were classified using a zero to five star rating, based on the WHO/AFRO quality improvement stepwise approach. The data provided is a trend analysis and has not been subjected yet to statistical significance analysis such as standard deviations and p-values based on data size.

### Ethical Clearance

The FHI360 Office of International Research Ethics (OIRE), North Carolina, USA, approved this project as exempt from further review (Project No: 577892-1).

## Results

**Figure 1:**
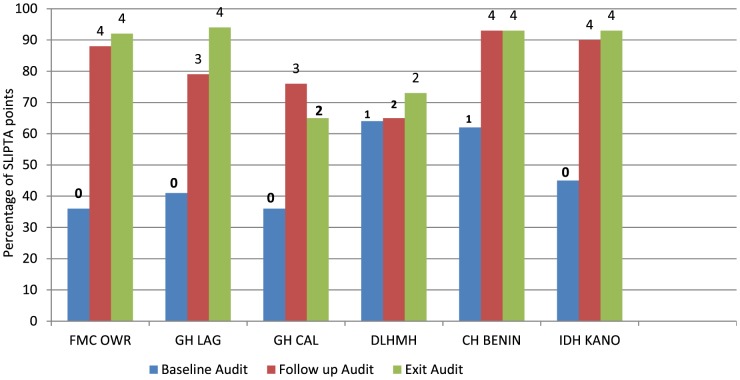
Performance of the various laboratories at baseline to exit audit as measured by the WHO-AFRO SLIPTA checklist. Numbers (0–5) on the bars represent the WHO/AFRO star rating. FMC: Federal Medical Center Owerri; GH: General Hospital Lagos; GH: General Hospital Calabar; DLHMH: Dr Lawrence Henshaw Memorial Hospital, Calabar; CH: Central Hospital Benin; IDH: Infectious Disease Hospital Kano,

Only two of the laboratories attained 1- star rating at baseline while the remaining four were on 0- star. During the follow up audit, one of the laboratory moved from o 1- star to 2-star, one moved from 1- star to 4- star, two from 0 to 3- star and the remaining two from 0 to 4- star rating. Four of the labs at exit audit were on 4- star and two on 2-star rating (Fig. 1). Five laboratories demonstrated a consistent and progressive improvement from baseline through the exit audit with a score of up to 132 points increase, representing an absolute gain of 61% in the most improved laboratory (FMC Owerri). One of the laboratories (GH Calabar) regressed with a score of 26 points decrease, representing an absolute loss of about 13% decrease between the follow up and exit audit.

**Figure 2:**
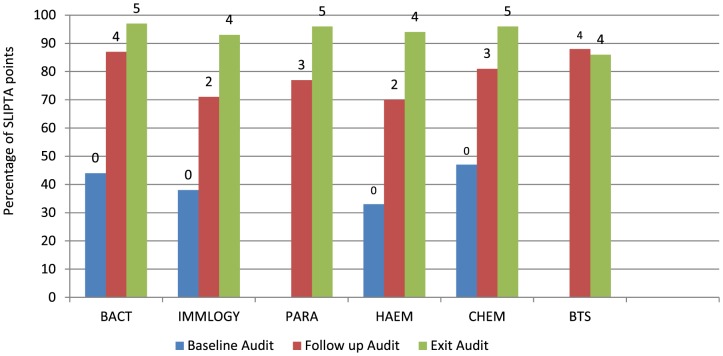
Performance of the various laboratory departments in General Hospital Lagos at baseline to exit audit as measured by the WHO-AFRO SLIPTA checklist. Numbers (0–5) on the bars represent the WHO/AFRO star rating. BACT: Bacteriology, IMMLOGY: Immunology & Serology, *PARA: Parasitology, HAEM: Haematology, CHEM: Chemistry, *BTS: Blood Transfusion Services, * did not participate in the baseline audit.

The audit result of the fully integrated laboratory (GH Lagos) also shows a progressive improvement across all the assessed sections with the exception of Blood Transfusion Services (BTS) (Fig. 2). The highest performance increase (65%) was recorded in Hematology unit between the baseline and exit audit while BTS regressed with a percentage of 14% between the follow up and exit audit (Fig. 2)

**Figure 3:**
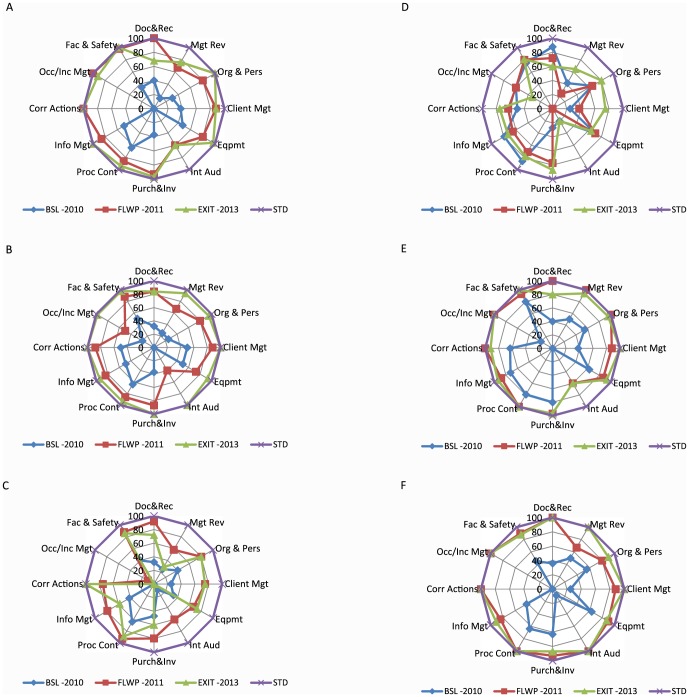
Performance of the various laboratories across the Quality Systems Essentials, as measured by the WHO-AFRO SLIPTA checklist from baseline to exit audit. Fig 3A: FMC Owerri; Fig 3B: GH Lagos; Fig 3C: GH Calabar; Fig 3D: DLHMH Calabar; Fig 3E: CH Benin; Fig 3F: IDH Kano (Doc & Rec: Document and Record, Mgt Rev: Management Review, Org & Pers: Organization & Personnel, Client Mgt; Client Management & Customer service, Eqpmt: Equipment, Int Aud: Internal Audit, Purch & Inv: Purchasing & Inventory, Proc Cont: Process Control and Internal & External Quality Audit, Info Mgt: Information Management, Corr Actions: Corrective Actions, Occurrence/Inc Mgt: Occurrence/Incidence Management & Process improvement, Fac & Safety: Facilities and safety).

The performance of the various laboratory across the 12 QSE following the WHO–AFRO SLIPTA checklist at baseline, follow up and exit audit is illustrated in Fig. 3 A–F. Continuous improvement was observed in each of the assessed QSEs in four laboratories (Fig. 3 A, B, E, and F). However in two other laboratories there was decline in some QSEs (Fig. 3 C and D). For example document and record, internal audit, information management, management review and purchase and inventory decline between follow up and exit audit (Fig. 3C). For the other site as shown in Fig. 3D, the QSEs document and record, information management and process control decline between baseline and follow up audits.

**Figure 4:**
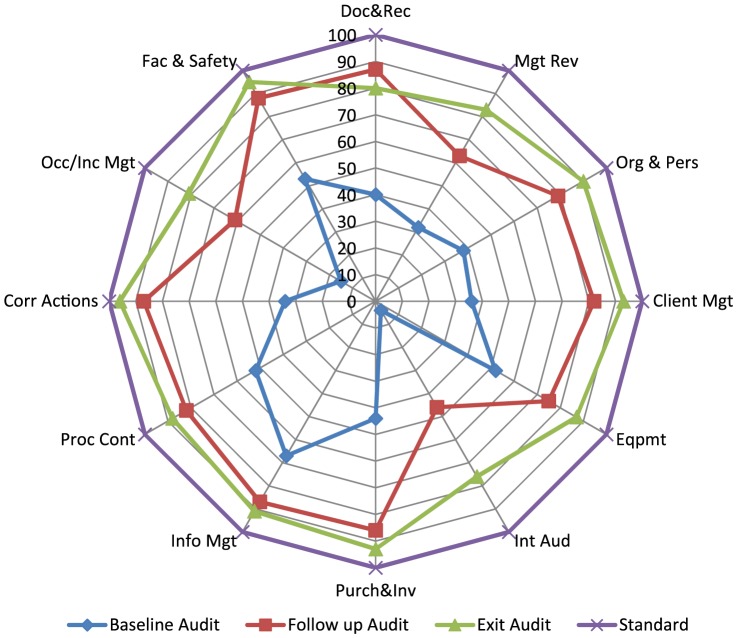
Average performance of all laboratories across the Quality Systems Essentials, as measured by the WHO-AFRO SLIPTA checklist from baseline to exit audit. (Doc & Rec: Document and Record, Mgt Rev: Management Review, Org & Pers: Organization & Personnel, Client Mgt; Client Management & Customer service, Eqpmt: Equipment, Int Aud: Internal Audit, Purch & Inv: Purchasing & Inventory, Proc Cont: Process Control and Internal & External Quality Audit, Info Mgt: Information Management, Corr Actions: Corrective Actions, Occurrence/Inc Mgt: Occurrence/Incidence Management & Process improvement, Fac & Safety: Facilities and safety).

The average performance of the six laboratories across the 12 QSE following the WHO–AFRO SLIPTA checklist from baseline to exit audit is illustrated in Fig. 4. Overall, there was a significant improvement in the assessed QSE over the entire period of intervention (Fig. 4). The two weakest elements at baseline; internal audit (4%) and occurrence/incidence management (15%) improved significantly with exit score of 76% and 81% respectively. While these two elements remained the most improved elements of the checklist, facility and safety was the major strength across board measured by the steady performance during the three phases of the audit exercise. The average performance at exit audit rated internal audit as the element with the lowest score (76%) while corrective action recorded the highest score of 96%.

## Discussion

The launch of the SLMTA training program [8] and SLIPTA process [9] has triggered a major interest in some African countries to strengthen and improve on clinical and public health laboratories services. The effort in Nigeria was coordinated centrally to support 23 selected laboratories in the pilot phase. Six of these laboratories are supported by FHI360 and the improvement process reported in this study is based on the six laboratories. Monitoring the performance of the six laboratories showed that only two had at least a 1-star status rating at baseline. This is comparable with an assessment involving 954 laboratories in Kampala, Uganda and 25 laboratories in Lesotho which reported that about 96% were at zero star [12] [13]. Although we did not specifically evaluate staffing level and mix in relationship to laboratory quality performance, it has been reported to be important based on a survey of laboratories in Kampala Uganda [12]. On the contrary, four of the laboratories in our study with adequate staffing had 0-star at baseline, unlike GH Benin with a 1- star rating having only four staff with two as laboratory scientist. It is worth noting that, Edo State (where GH Benin is located) laboratory leadership have always manifested keen interest in laboratory quality issues even before the WHO laboratory accreditation champagne.

One year after baseline assessments laboratories showed an overall marked improvement with five of the six laboratories ranked at 4-stars on average. At exit audit, about 26 months after the baseline audit, four of the labs were on 4-star, one on 3-star and one on 2-star rating. As a whole, there was measurable improvement in all the assessed QSE from baseline through the exit audit (Fig. 4).

Technical assistance by FHI360, in terms of infrastructural upgrade, capacity building, improvement projects, follow up visits, mentoring as well as advocacy has largely resulted to the measurable quality improvement and the success of the program in the six laboratories. In addition, commitment from facility technical staff, some level of ownership and support by facility management, State and Federal Ministry of Health was crucial to the success. For example, the from the management of GH Lagos management carried out infrastructural and equipment upgrade and sponsored two additional participants to attend SLMTA workshop. FMC Owerri management also provided safety kits like eye shields and goggles as part of their committed and support to the process. Similar success in implementing the SLMTA program was reported in Lesotho and was largely attributed to strong leadership and ownership by the government [13].

Despite the consistent and progressive improvement observed in five of the laboratories one laboratory recorded a decline of about 14% between the follow up and exit audit (Fig. 1). As observed by FHI360 laboratory technical officers supporting the facility, this is a scenario where the facility management and State Ministry of Health have not been able to put a committed and dedicated team in place for the program. Sustainability of this program is questionable after the withdrawal of technical support from implementing partners. Similar concerns perceived within African countries have led to a plea for the government to take greater responsibility to handle the challenges facing public health laboratory as donor support is currently threatened by global recession, economic hardship and other competing demands [14]. A decline in some QSE scores between baseline and follow up audit in one laboratory (Fig. 3 D), was probably because the baseline assessment was done by a TB laboratory support consultant alone unlike the follow up audit done by certified SLMTA auditors. Besides this one scenario, inter-rater reliability in use of the SLMTA checklist was maximized through the use of trained SLMTA auditors (2 to 3 per case), consistent documentation of comments to substantiate audit score and a consensus before release of final score.

The strongest QSE element observed across all facilities during this process is facility & safety; this was evidenced by the steady performance during the 3 phases of the audit exercise. The two weakest elements at baseline; internal audit (4%) and occurrence/incidence management (15%) is attributed to the lack of basic understanding of these important components of QMS, as well as the poor attitude towards documentation by most laboratory personnel. With targeted training and mentoring processes, performance improved significantly with exit score of 76% and 81% respectively. However, internal audit was the element with the lowest score at exit audit and this critically demands a targeted and specific type of mentorship in order to institutionalize a functional internal audit system.

Although at national level, FHI360 was considered to be providing support to six laboratories, GH Lagos alone consist of six laboratories (comprising of six specialized department). Efforts to support each department in implementing the QMS, is equivalent to the support for each of the other five separate laboratories. However, for GH Lagos we fully capitalized on grouping and leveraging resources during planning and implementation of the process. The overall progressive improvement observed at GH Lagos within the different departments was remarkable. This was also recognized internationally with an award from the African Society of Laboratory Medicine (ASLM) for second place in “Best Laboratory Practice” during the maiden edition of the ASLM conference in December 2012 [15].

In principle, the SLMTA implementation for laboratory support to be ready for international accreditation is estimated at 18^th^ months [9]. Our implementation period was longer largely due to the few numbers (only 6) of SLMTA trained auditors in Nigeria that are responsible for audits and technical support for 23 pilot sites and limited technical know-how of the process by the other stakeholders with few experienced mentors. Thus there is usually a long gap between training and execution of intervention in the facility meant to transform classroom exposure to actual bench implementation.

Several challenge to integrate the SLMTA process into everyday laboratory work exist. There were attitudinal issues with staff, demonstrated through reluctance to cooperate and comply as they consider QSM implementation as an additional burden (especially paper work burden) and not part of routine work. Implementation of SLMTA is a long and continuous process and needs proper planning, schedules and dedicated personnel to follow this through. It was also difficult to adhere to assessment schedules as disruptions arising from strike action, security challenges as well as auditor availability. Due to resource constraint and the limited number of slots available in the SLMTA Nigeria program, only a maximum of 21 persons were formally trained with the responsibility of stepping down the training to the rest of the facility laboratory team and lead the implementation process at the facility level. The quality of the step down trainings could not be guaranteed.

One of the direct positive impacts of the SLMTA process as observed in some of the six laboratories is the emergence of trained facility staff with required skills for the implementation of QMS. They are now available to serve as peer mentors to initiate and support the process of other laboratories being considered in the scale up phase. Over time with more competent and experienced mentors along with a structured mentorship program, the QMS program will likely be accelerated and improved as experienced in Lesotho [16] [17].

We have demonstrated that implementing the SLMTA process to improve laboratory quality systems is feasible in Nigeria through top-bottom advocacy, management involvement, assessment, gap analysis, capacity building, training and mentorship.

## Conclusions

The SLMTA program improved performance as determined by the audit results. Overall these efforts resulted in remarkable improved star ratings and QSE scores which may be indicative of reliable test results and improved patient care. Frequent internal audits can ease the accreditation process. We recommend further improvement and participation in a formal International accreditation scheme. This is a good reason to continue to roll out the SLMTA program, as this will bring about continuous and sustainable laboratory quality improvement. Capacity building, improvement projects, follow up visit, sustained mentorship, advocacy, commitment among all stake holders are vital to maintain and improve on these results.
